# Gut Microbiota Dysbiosis, Oxidative Stress, Inflammation, and Epigenetic Alterations in Metabolic Diseases

**DOI:** 10.3390/antiox13080985

**Published:** 2024-08-14

**Authors:** Hamid Mostafavi Abdolmaleky, Jin-Rong Zhou

**Affiliations:** 1 Nutrition/Metabolism Laboratory, Beth Israel Deaconess Medical Center, Harvard Medical School, Boston, MA 02215, USA; 2 Department of Medicine (Biomedical Genetics), Boston University Chobanian & Avedisian School of Medicine, Boston, MA 02118, USA

**Keywords:** gut dysbiosis, microbiota, microbiome, oxidative stress, inflammation, epigenetic, transgenerational, metabolic diseases

## Abstract

Gut dysbiosis, resulting from an imbalance in the gut microbiome, can induce excessive production of reactive oxygen species (ROS), leading to inflammation, DNA damage, activation of the immune system, and epigenetic alterations of critical genes involved in the metabolic pathways. Gut dysbiosis-induced inflammation can also disrupt the gut barrier integrity and increase intestinal permeability, which allows gut-derived toxic products to enter the liver and systemic circulation, further triggering oxidative stress, inflammation, and epigenetic alterations associated with metabolic diseases. However, specific gut-derived metabolites, such as short-chain fatty acids (SCFAs), lactate, and vitamins, can modulate oxidative stress and the immune system through epigenetic mechanisms, thereby improving metabolic function. Gut microbiota and diet-induced metabolic diseases, such as obesity, insulin resistance, dyslipidemia, and hypertension, can transfer to the next generation, involving epigenetic mechanisms. In this review, we will introduce the key epigenetic alterations that, along with gut dysbiosis and ROS, are engaged in developing metabolic diseases. Finally, we will discuss potential therapeutic interventions such as dietary modifications, prebiotics, probiotics, postbiotics, and fecal microbiota transplantation, which may reduce oxidative stress and inflammation associated with metabolic syndrome by altering gut microbiota and epigenetic alterations. In summary, this review highlights the crucial role of gut microbiota dysbiosis, oxidative stress, and inflammation in the pathogenesis of metabolic diseases, with a particular focus on epigenetic alterations (including histone modifications, DNA methylomics, and RNA interference) and potential interventions that may prevent or improve metabolic diseases.

## 1. Introduction

Metabolic diseases are medical conditions caused by abnormal biochemical reactions in cellular metabolism during which the body converts food into energy and other essential substances required for a healthy life. Metabolic diseases often have syndromic features and result from genetic or epigenetic aberrations, enzymatic defects, or dysregulation of metabolic pathways, leading to the development of an array of symptoms related to body growth, energy generation, and toxin elimination. The incidence of metabolic diseases increases with aging. However, several environmental factors, including food content, infectious diseases, gut dysbiosis, exposure to toxins or contaminants, lack of physical activity, and chronic psychological stress, also contribute to the development of metabolic diseases [1,2]. Among genetic factors, mitochondrial disease and higher levels of cholesterol and lipids are well-known contributing factors [3]. Among non-genetic factors, gut dysbiosis, which depends on gut microbiota structure, is considered one of the most important factors in recent years [4].

The mechanisms through which an inappropriate diet, lack of physical activity, and exposure to toxins/contaminants induce a metabolic disease have been primarily described in the last few decades [5]. However, the details of gut microbiota interference in metabolic diseases pertaining to epigenetic modifications have yet to be adequately described. In this work, we review the most recent findings related to the impact of gut microbiota in metabolic diseases and underlying mechanisms through which gut dysbiosis, by inducing inflammation and oxidative stress through epigenetic alterations, may contribute to the disease pathogenesis in common metabolic diseases. To explore this subject, we first offer a brief overview of gut microbiota structure, its development, and its interaction with food content in a healthy state. We then present recent research findings from the past five years that examine the link between gut dysbiosis and inflammation, oxidative stress, and epigenetic alterations in metabolic diseases. This study aims to fill the knowledge gap regarding how gut microbiota influences metabolic diseases such as diabetes, obesity, cardiovascular disease, and non-alcoholic fatty liver disease (NAFLD), which can transfer to the next generation involving epigenetic mechanisms. Additionally, the study explores potential therapeutic interventions, such as dietary changes, probiotics, fecal microbiota transplantation, and physical exercise, which may mitigate oxidative stress and inflammation associated with metabolic diseases through epigenetic mechanisms. These interventions offer new avenues for treating these increasingly common diseases in the context of modern industrialized living conditions.

## 2. Gut Microbiota Structure and Gut Microbiome Development in Mammals

There is a dynamic ecosystem inside our digestive system where over 100 trillion microbial elements, collectively containing almost 3 million genes (roughly 100-fold more than human genes), are living throughout our life in the gut [6]. While gut microbiota consists of >2000 different types of bacteria, viruses, fungi, and protozoa [7], the total number of gut microbes surpasses that of human body cells (30–40 trillion), and they produce numerous metabolites that are largely unknown [8]. A large fraction of these microbial metabolites can pass through the intestinal wall/barrier and enter the liver or other tissues via blood circulation. A healthy gut can prevent the entry of toxins and harmful metabolites into blood circulation while permitting the entry of useful metabolites [9,10]. This gut barrier has developed and evolved over several millions of years in interaction with gut microbiota in a species-specific manner. However, the species’ genetic structure, food composition, age, geographical conditions, etc., also intervene. The gut ecosystem is an ocean of various components interacting with each other and the host, particularly the intestinal wall and its resident immune cells (the intestinal dendritic cells), which protect the host against infiltration/intrusion of bacterial elements or their antigens/toxins into the blood circulation [11,12,13,14]. The functionality of this protective intestinal wall can be disrupted by many external or internal factors, such as infection, toxins, contaminants, food components, aging, oxidative stress, inflammation, metabolic diseases, etc. However, chronic disruption of the intestinal wall’s protective functions can also induce metabolic diseases. Concerning foreign antigens and infectious elements, a continuous and efficient functionality of the dendritic cells and macrophages of the intestinal wall is very important [12,13,15]. While any tissue directly exposed to the external environment possesses a type of dendritic cell that surveys the tissue for foreign elements, this protective mechanism is more sophisticated in the intestinal wall.

Evidence shows that the interaction between gut microbiota and the host intestinal wall starts even before birth, as newborns’ meconium is not germ-free at birth [16]. Although a more recent study reports the sterility of meconium at birth [17], it is still conceivable that during pregnancy, some maternal gut microbes may pass through the intestinal wall and end up homing in the fetus’s gut, potentially leading to health-altering outcomes [18]. Furthermore, prenatal exposure to environmental pollutants, such as di-(2-ethylhexyl)-phthalate (DEHP), widely used in the plastics industry, may result in gut microbiota dysbiosis and metabolic syndrome in male offspring, which is attenuated by thiamine [19]. In mice, in utero exposure to arsenic may also decrease the abundance of gut *Firmicutes*, which could have a functional impact on genes related to insulin signaling and NAFLD in offspring [20].

Human gut bacteria are generally detectable almost 16 h after birth [21]. The newborn acquires most of the gut bacteria from the mother, but paternal and sibling microbiota also contribute to the infant microbiota through direct contact, such as touching, kissing, and sharing the same living environment [22]. There is evidence that S100A8 and S100A9 calcium-binding proteins, abundant in human breast milk, further regulate the development of intestinal microbiota and the immune system reaction in neonates [23].

Following the initial development, the gut microbiota and its metabolome exhibit diurnal rhythmicity that provokes the oscillation of the host transcriptome, metabolic state, and epigenetic programming [24]. However, antibiotic treatment could perturb the brain’s circadian metabolic cycles, primarily involving the suprachiasmatic nucleus [25]. Another study revealed that gut microbes that produce short-chain fatty acids (SCFAs) orchestrate intestinal epithelial circadian rhythms, and histone deacethylase (HDAC) inhibitors can reduce this effect [26]. Interestingly, butyrate, one of the most potent gut-produced SCFAs, also regulates circadian clock genes in muscle tissue and attenuates high-fat diet-induced obesity. This is associated with increased gut abundance of *Firmicutes* bacteria, increased histone acetylation of the muscle tissue, and upregulation of circadian clock genes [27]. Along with alterations in response to circadian rhythms, the gut microbiota composition is altered in different seasons due to dietary shifts. For instance, a longitudinal examination of the stool microbiome from the Hadza hunter–gatherers of Tanzania, whose diet relies on seasonal foraging for wild plants, fruits, tubers, and hunting, revealed that while some taxa become undetectable in a specific season, they reappear again in other seasons. Surprisingly, during the cyclic disappearance of these taxa, their microbiota profiles trend towards the microbiome composition of industrialized societies [28].

There is also evidence that moderate-altitude alteration affects gut microbiome composition (increases *Bacteroidetes* and decreases *Proteobacteria* abundance) in humans, positively affecting fasting blood glucose level and serum metabolome. Furthermore, fecal transplantation from these individuals could mitigate the diverse metabolic effects of a high-fat diet in mice [29].

Aging is another determinant of gut microbiome alterations, immune cell dysfunction, and the increased incidence of metabolic diseases, along with an increase in reactive oxygen species (ROS) production, epigenetic alterations, and mitochondrial dysfunction [30,31,32]. In general, there is a trend toward decreased microbial diversity and increased abundance of pathogenic bacteria as we age [33,34]. Brunt et al. uncovered that age-related gut microbial alterations are linked to gut dysbiosis, and plasma level of trimethylamine N-oxide (TMAO, a gut-derived metabolite) is higher in aged versus young mice. Remarkably, non-absorbent antibiotic treatment was associated with increased antioxidant enzyme expression, decreased oxidative stress and plasma TMAO levels, and improved arterial endothelial dysfunction [35], indicating that gut microbiota and ROS are involved in age-related metabolic diseases. A recent study has also shown that specific microbiome alterations (e.g., relative abundance of *Actinobacteria*, *Proteobacteria*, and *Collinsella aerofaciens* in males and *Bacteroidetes* and *Dorea longicatena* in females) are linked to the acceleration of epigenetic aging, while physical fitness, “exercise-related parameters”, and bacterial species with anti-inflammatory effects (e.g., *Fusicatenibacter saccharivorans* and *Anaerostipes hadrus*) are protective [36]. Regarding the influence of physical exercise, a systematic review of 28 longitudinal human studies found that moderate- to high-intensity exercise (30–90 min three times per week or 150–270 min per week) for 8 weeks could change the gut microbiota composition, increasing the abundances of *Roseburia*, *Lachnospira*, *Dorea*, and *Ruminococcus* in response to aerobic exercise. However, intensive exercise (90 min and more than five times per week) may have negative effects [37]. A 12-week physical exercise program in pediatric patients with obesity also modulated gut microbiota (e.g., increased *Roseburia, Blautia*, and *Dialister,* a profile that resembles that of healthy children) and reduced inflammation and *NLRP3* expression [38], where the NLRP3 inflammasome activation is linked to inflammation and ROS generation [39].

## 3. The Gut Microbiome and Diet Interaction

While diet is one of the significant determinants of gut microbiota structure, experimental studies have shown that a high-fat or high-sugar diet can rapidly alter gut microbiome composition in less than four days. This alteration could be reversible by reestablishing the prior diet, but the memory of previous exposure is also maintained [40]. In another study, a 12-week high-sugar and high-fat diet induced obesity, increased *Firmicutes* and *Actinobacteria*, and decreased *Bacteroidetes* phyla associated with altered expression of hypothalamic neuropeptides (e.g., Npy, Gal, and Galr1), anxiety, and impulsive behavior [41]. Meanwhile, a different study in mice revealed that fiber, rather than fat, influences gut microbiota structure. In this study [38], Morrison et al. have shown that the difference in the transition from a regular diet to a high-fat diet is the lack of fiber in a high-fat diet. To elaborate on this hypothesis, they used high- and low-fat diets with the same amount of soluble fiber and reported no significant difference in the gut microbiota composition. However, using a low- vs. high-fiber diet led to a decrease in the abundance of *Bacteroidetes* and an increase in the abundance of *Clostridia* and *Proteobacteria* [42].

In a human study, it has been shown that short-term consumption of animal-based versus plant-based foods is capable of rapidly altering gut microbiota composition. This change is associated with a decrease in the abundance of *Firmicutes*, which metabolize plant-based polysaccharides, and an increase in the abundance of bile-tolerant bacteria such as *Alistipes, Bacteroides*, and *Bilophila*, the latter of which can induce inflammatory bowel diseases [43]. Another human study revealed that, although dietary pattern alteration from animal-based to plant-based food can change gut bacterial structure within 24 h (shifting from *Bacteroides*, associated with animal food, to *Prevotella*, linked to carbohydrates), long-term dietary modification is essential to consolidate the gut bacteria enterotypes [44]. The diet promotes the growth of beneficial bacteria, increases microbial diversity, and enhances the production of SCFAs, which are crucial for gut health. Furthermore, experimental evidence suggests that the gut microbiota not only contributes to the synthesis and absorption of nutrients and vitamins [45] but also determines the uptake of energy from food. For instance, it has been shown that, while obesity is linked to a relative abundance of *Bacteroidetes* and *Firmicutes* and the microbiome of individuals with obesity harvests energy from the diet more efficiently, the transfer of their gut microbiota to germ-free mice increases the mice’s total body fat [46].

## 4. Gut Dysbiosis and ROS Production

Gut dysbiosis, resulting from an imbalance in the gut microbiome, can induce excessive production of ROS, leading to inflammation by disrupting gut barrier integrity, activation of the immune system, and alteration of metabolic pathways. As gut dysbiosis alters gut microbial metabolites that may induce inflammation, ROS production, and epigenetic alterations [47], inflammation-induced ROS can also intensify gut dysbiosis [48,49]. Experimental studies in mice have shown that host ROS production alters the gut microbiota species diversity and gut microbiome composition [50]. On the other hand, probiotics that alleviate gut dysbiosis can mitigate gut ROS production, thus reducing ROS-induced inflammation [51,52]. Similarly, dietary polyphenols can suppress gut dysbiosis by scavenging ROS and increasing the abundance of the beneficial *Akkermansia muciniphila* bacteria, which is reduced in obese mice, and whose abundance is associated with a decrease in gut extracellular ROS levels. Notably, among different antioxidants, such as vitamin C, β-carotene, and grape polyphenols, the latter explicitly reduces gut ROS and promotes the growth of *Akkermansia muciniphila* due to its lower bioavailability [53]. It is also important to note that, despite the harmful effects of ROS on gut health, ROS plays a significant role in stem cell proliferation through interactions with the gut microbiota in colon epithelial cells. In this potentially evolutionary adaptive process, gut microbiota activates toll-like receptors, which stimulate *NOX1* expression, leading to ROS production, activation of epidermal growth factor receptor (EGFR) signaling, and cell proliferation to preserve colon homeostasis [54].

## 5. Metabolic Impacts of Gut Dysbiosis Involving Epigenetic Mechanisms

**Figure 1:**
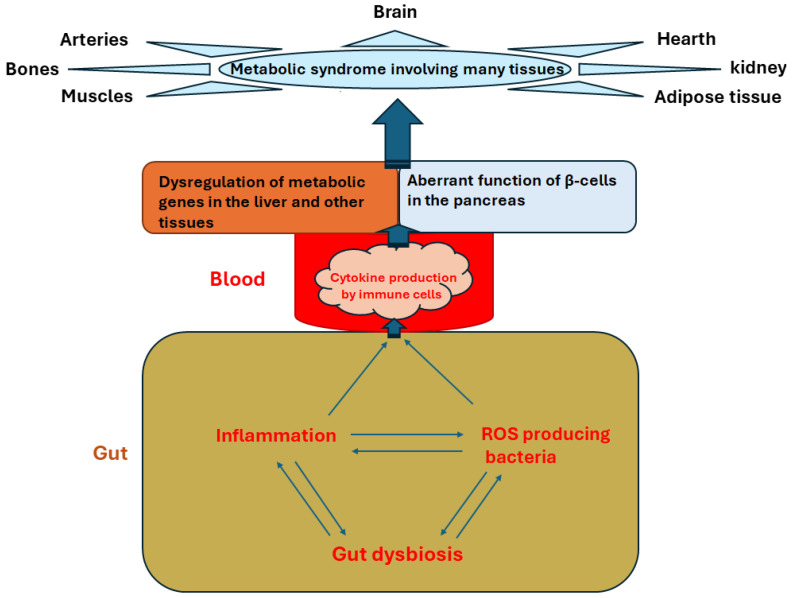
The cascade of events through which gut dysbiosis induces metabolic diseases. While gut dysbiosis increases inflammation, it also expands the presence of ROS-producing bacteria, which further exacerbates inflammation. Gut dysbiosis is intensified by both ROS and inflammation, which reduces intestinal wall integrity and allows bacterial antigens and toxic materials to penetrate gut cells and enter the bloodstream, further amplifying ROS production. This leads to immune cell activation and the production of inflammatory cytokines. These inflammatory cytokines affect the function of metabolic genes in the pancreas, liver, and other tissues, leading to the dysfunction of different tissues observed in metabolic syndrome.

It has been shown that microbiota metabolites interact with hundreds of G protein-coupled receptors [55] involved in metabolic diseases [56,57]. Therefore, it is not surprising that gut microbial dysbiosis is associated with several metabolic diseases, like type 2 diabetes, atherosclerosis, and NAFLD [58,59,60]. In addition to the contribution of ROS to inflammation and the disruption in gut barrier integrity mentioned above, this relationship could be due to altered bile acid metabolism and the pleiotropic effects of metabolites and inflammatory cytokines produced by the gut microbiota [61]. Figure 1 illustrates the cascade of events through which gut dysbiosis, associated with an imbalance in ROS-generating and ROS-decreasing bacteria, contributes to inflammation and the development or progression of metabolic syndrome.

In human studies, an increase in taurine/hypotaurine and lipoic acid metabolism due to the reduced abundance of *Ruminococcus* and *Bifidobacterium*, along with lower vitamin D intake, has been observed in individuals at higher risk for cardiovascular diseases. However, a higher intake of vitamins D and A, as well as protein and monounsaturated fat, could increase the abundance of the *Ruminococcus* genus [62]. In non-obese type 2 diabetic MKR mice (which carry a muscle-specific mutation of the *Igf-1* receptor gene), gut dysbiosis is associated with reduced abundances of butyrate-forming bacteria and *Bacteroides fragilis*, which ferment complex carbohydrates for SCFA production. This leads to HDAC3 (histone deacetylase 3) activity dominance, increased colon permeability, ROS production, NOX4 (a protein catalyzing superoxide generation from molecular oxygen), and IL-1β levels but reduction in IL-10 and IL-17α levels. In contrast, treatment with butyrate (a SCFA which inhibits HDACs) could restore normal levels of NOX4 and IL-1β [63].

Regarding the mechanisms of SCFA actions, while the general viewpoint of the scientific community is that SCFAs inhibit HDAC activities, one study shows that at least butyrate and propionate (but not acetate), after being converted to acyl-CoAs, activate the acetyltransferase p300 enzyme, which subsequently induces histone/protein acetylation [64]. However, acetate, through suppression of the increased circulating and hypothalamic HDAC5 in fructose-induced type 2 diabetes mellitus (T2DM) rats, may attenuate metabolic syndrome in the affected animals [65]. Meanwhile, another study reported that despite transcriptomic alterations, the global hepatic histone acetylation level is not affected by antibiotic-induced gut microbiota depletion, which is associated with the lack of SCFA production by the gut microbiota. Furthermore, SCFA supplementation could not affect global hepatic histone acetylation in the absence of gut microbiota [66], suggesting that other mechanisms or tissues may be involved in the protective effects of SCFAs in metabolic diseases. To this end, a study showed that, while gut microbiota dysbiosis of children with new-onset type 1 diabetes was associated with decreases in butyrate production and bile acid metabolism and an increase in lipopolysaccharide (LPS) biosynthesis, butyrate could protect pancreatic islet structure and function and enhance insulin 1 and 2 (*Ins1* and *Ins2*) gene expression levels in mice [67].

Interestingly, the gut microbiota of these children transplanted to germ-free mice could increase fasting glucose levels and decrease insulin sensitivity. Additionally, LPS was shown to increase pancreatic inflammation with destructive effects on the structure and function of pancreatic islets [67]. Trichostatin-A, another HDAC inhibitor, could also mitigate weight loss and intestinal injury by increasing the gut *Firmicutes*/*Bacteriodetes* ratio and reducing the expression of *NF-κB*, *Cox-2*, and *iNOS* in the intestine of 15 Gy-irradiated mice [68]. In addition to these genes, *Mmp-12* and *Fabp4* play significant roles in small intestine-mediated metabolic diseases. MMP-12, as a transcriptional factor, promotes *Fabp4* transcription through an epigenetic mechanism in the small intestine and mediates high-fat diet-induced obesity. However, small intestine-specific knockdown of this gene improves metabolic disorders and intestinal homeostasis, decreases inflammation, lipid transportation, and bile acid reabsorption, and restores gut microbiota composition [69].

In addition to SCFAs, other gut microbiota-produced metabolites, such as choline (the main source of methyl groups), and some vitamins (in particular, vitamin B12 and folate) can also act as substrates or cofactors for enzymes involved in histone modifications and DNA methylation [70,71,72]. Lactate, in addition to being a byproduct of glycolysis, which is affected in metabolic diseases, is another product of the gut microbiota that induces gene expression through histone lactylation [73].

## 6. Gut Microbiome, Inflammation, ROS, and DNA Methylome Interactions

A multi-omics study revealed that host microbiota and DNA methylome interactions affect gene functions linked to inflammation, metabolic diseases, and oxidative stress in the intestinal wall and blood cells in Crohn’s disease [74]. Still, several other factors may contribute to these interactions. For example, hyperglycemia, which induces ROS and impairs antioxidant defense systems [75], impacts gut microbiome structure and metabolism and intensifies drug-induced dysbiosis and energy catabolism [76]. Nevertheless, the gut microbiome is also involved in intestinal maturation by directing postnatal DNA methylation of glycosylation genes at its 3′ CpG islands in intestinal stem cells [77]. Furthermore, whole-genome bisulfite sequencing in germ-free versus conventionally raised mice uncovered that the exposure of the intestinal epithelium to commensal microbiota directs TET2/3-dependent DNA methylation alterations at the regulatory elements of a set of genes to maintain intestinal homeostasis. While this microbiota-induced epigenetic reprogramming might be essential for maintaining proper intestinal homeostasis, in acute gut inflammation (induced by dextran sodium sulfate, DDS), the exposure of the intestinal epithelium to microbiota leads to aberrant DNA methylation and chromatin modifications at regulatory elements, resembling those observed in colitis [78]. Similarly, early-life inflammatory stressors could trigger a sustained epithelial injury and increase gut permeability due to downregulation of E-cadherin expression (an epithelial junction protein), mediated by increased expression of miR-155, secondary to its promoter histone hyperacetylation [79]. However, compounds with antioxidant and anti-inflammatory properties (e.g., cinnamon) may have protective effects by increasing E-cadherin-2 expression [80]. Selenium-containing amino acids, such as selenocysteine and its derivative selenocystine, could also ameliorate DSS-induced oxidative stress and intestinal inflammation in mice [81].

**Figure 2:**
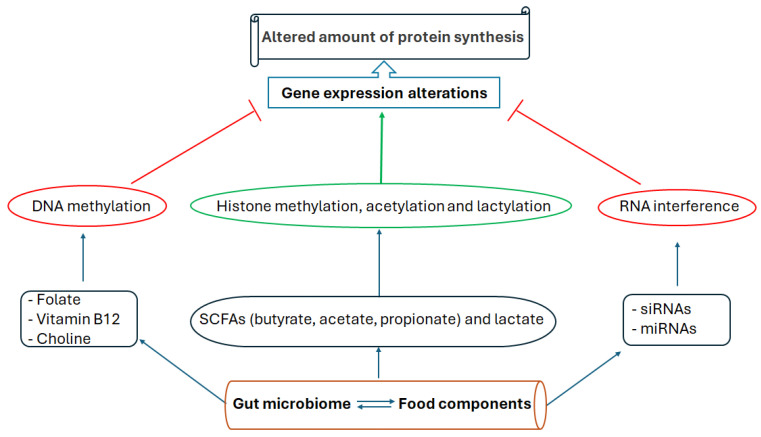
The cascade of events where gut microbiota and food interaction produce different metabolites, vitamins, and bioactive compounds as well as miRNAs. The gut microbiota products influence DNA and histone methylation, or histone acetylation and lactylation, which in turn affect gene expression levels. Additionally, miRNAs and siRNAs produced by gut microbiota or cells of the digestive tract induce RNA degradation or inhibit viral RNA and transposons, respectively. While DNA methylation and RNA interference commonly inhibit gene expression, histone methylation has dual functions (depending on the methylation of specific amino acid residues of the histone tail proteins). Histone acetylation and lactylation generally stimulate gene expression. Arrows indicate directionality, while T-shaped marks indicate suppression.

In humans, a correlational gut microbiota and DNA methylation study in individuals undergoing a 3-month behavioral weight loss procedure uncovered that *Ruminiclostridium* abundance was correlated with DNA methylation of *COL20A1*, *COL18A1*, and *NT5E* genes at the baseline. A three-month intervention changed this pattern, and *Ruminococcus gnavus* was positively correlated with DNA methylation of the *CA3* gene. Additionally, the abundance of *Akkermansia* was inversely correlated to DNA methylation of *CRYL1* (implicated in antioxidant defense mechanisms), *C9* (involved in immunity and inflammation, which is linked to ROS), as well as *GUSB* and *GMDS* (involved in carbohydrate metabolism and glycosylation processes, respectively). The same was true for *Lachnospiraceae* UCG-001, which had an inverse correlation with DNA methylation of *NR5A2* (involved in the oxidative stress response and redox regulation), *HES1* (also involved in the oxidative stress response), as well as *PCLO*, and *LRAT* genes. Since none of these correlations had relationships with dietary intake, the authors concluded that “microbes linked to mucin degradation, SCFA production, and body weight are associated with DNA methylation of phenotypically relevant genes” [82]. Another human study of individuals affected by metabolic disease showed that allogeneic (vs. autologous) fecal microbial transplant (from lean donors) leads to a gut microbiota shift and modulation of the plasma metabolome and the epigenome of blood mononuclear cells. Specifically, *Prevotella* abundance was associated with DNA methylation of the *AFAP1* gene that affects mitochondrial function and insulin sensitivity [83]. Figure 2 illustrates how the interaction between the gut microbiota and food produces various components or substrates that can influence different mechanisms of epigenetic modulation, leading to changes in gene expression and protein synthesis.

## 7. Transfer of Gut Microbiota-Related Metabolic Diseases to the Next Generation through Epigenetic Mechanism

In human studies, it has been shown that the neonatal blood metabolome correlates with the maternal fecal metabolome and that altered gut microbiota in maternal gestational diabetes mellitus may increase the risk of neonatal inborn metabolic errors [84]. In a more recent study, Duan et al. reported that antibiotic-induced maternal gut dysbiosis impacts the embryonic development of the enteric nervous system (ENS) by altering a signaling pathway involving propionate (a HDAC inhibitor) and *Gpr41* (a gene encoding a receptor for SCFAs), *Gdnf*, *Ret*, and *Sox10* genes in mice [85]. The ENS is a key component of the gut–brain axis, significantly influencing the body’s metabolic state. It regulates gastrointestinal functions and interacts with the gut microbiota to affect energy balance and nutrient absorption, impacting metabolic health [86,87].

Several other lines of evidence indicate that gut microbiota-induced metabolic dysfunctions can be transferred to the next generation through epigenetic alterations. As an introduction to this topic, it is important to note that a recent landmark study has demonstrated that induced epigenetic modifications in mouse embryonic stem cells—specifically, the addition of methyl groups to the CpGs of the promoter regions of two metabolic genes, *Ldlr* and *Ankrd26*—can suppress the expression of these genes. Remarkably, both the DNA methylation marks and the associated phenotypes, such as obesity, were found to be heritable, transferring to four subsequent generations of mice [88]. In relation to gut microbiota involvement in this context, a previous study by Romano et al. uncovered that, as some bacteria utilize choline, which is required for DNA methylation in mammals, mice on a high-fat diet and possessing elevated levels of choline-consuming bacteria are at a greater risk of metabolic diseases associated with global DNA methylation alterations in both the affected mice and their offspring, along with behavioral changes [89]. Maternal consumption of a Western-type diet during pregnancy in baboons could also affect the offspring’s microbiome. This type of diet not only alters the maternal gut microbiome (increase in *Clostridiales* and *Lactobacillales* abundance and decrease in alpha diversity) and lipid metabolism, along with increasing inflammation and ROS, but it also induces epigenetic changes in the placenta (miR-182-5p and miR-183-5p downregulation) and the offspring’s liver (miR-204-5p and miR-145-3p down-regulation), accompanied by dyslipidemia and systemic inflammation [90]. In another study in mice, Kimura et al. demonstrated that maternal microbiota during pregnancy shapes the offspring’s metabolic system, as SCFAs of the maternal microbiota direct intestinal, pancreatic, and neuronal cell differentiation mediated by embryonic SCFA receptors (GPR41 and GPR43), helping an efficient postnatal energy homeostasis. However, the offspring of germ-free pregnant mice exhibit increased susceptibility to metabolic syndrome [91], further confirming that maternal microbiota contributes to the maturation and functionality of the offspring’s metabolic system, mediated by the production of SCFAs by the maternal gut microbiome. Notably, in adult mice, similarly, SCFAs from a high-fiber diet or SCFA-producing microbiota are cardioprotective, mediated by the SCFA receptors GPR43/GPR109A, with important roles in the body metabolic and anti-inflammatory ROS responses [92].

A maternal low-protein diet during lactation also alters the gut microbiome in female F1 offspring, associated with diminished early-life growth rate and metabolic health, which can be transmitted to the following two generations. The oocytes of the F1 generation also exhibit DNA methylome alterations, which are partly transmitted to the oocytes of the F2 generation [93].

It has been shown that a paternal preconception diet (high-protein vs. high-fat/sucrose) also has protective metabolic effects in rats’ offspring by improving insulin sensitivity, circulating satiety hormones, and intestinal SCFA production. This was associated with altered intergenerational *Dnmt1* and *Dnmt3b* expression, an increase in the paternal bacterial diversity, and higher abundances of *Bifidobacterium*, *Akkermansia*, *Bacteroides* and *Marvinbryantia* in their male or female adult offspring [94]. In another study, supplementing a cocktail of methyl donors to a paternal high-fat/sugar diet in rats improved both paternal and offspring metabolic health and gut microbial signature, which was associated with decreased expression of *Dnmt3a* in paternal adipose tissue and miR-34a in hepatic tissue, upregulation of miR-24, miR-33, miR-122a, and miR-143 in adult male offspring, and downregulation of miR-33 in female offspring [95]. A paternal oligofructose-supplemented diet in rats could also induce changes in both paternal and offspring metabolic states and gut microbiota, such as increased abundance of *Bacteroidetes* in female and *Christensenellaceae* in male offspring [96].

In a more recent study in mice, preconception paternal gut microbiota perturbation by non-absorbent antibiotics has been linked to growth restriction, decreased birth weight, and premature mortality in their offspring. This is mediated by alterations in the testis histology, testicular metabolites, leptin signaling, and sperm small RNAs, mediating placental insufficiency in utero [97]. Harris et al. have also shown that in germ-free and T cell-deficient mice, the resulting defect in sebum secretion carries on to the next generations “despite microbial colonization and T cell repletion”. Interestingly, these trans-generationally inherited phenotypes are observed in progenies conceived by in vitro fertilization using the sperm and eggs of germ-free mice, indicating the involvement of non-genetic [likely epigenetic] inheritance [98]. A paternal Western-type diet in mice also indices phenotypic, gut microbiota, and behavioral changes in F1 offspring, such as higher body weights, associated with an increase in the gut abundance of *Actinobacteria*, preference for the Western type of food, and increased dominant behavior in male mice [99]. Paternal exposure to inorganic arsenic, known to induce widespread epigenetic alterations [100], could also lead to inter- and transgenerational metabolic and gut microbiome changes in F1, F2, and F3 male offspring [101].

## 8. Dietary and Probiotic Interventions to Modulate Gut Microbiome, ROS, and Metabolic Diseases

As mentioned before, diets and dietary compounds (commonly considered and prebiotics) play major roles in modulating gut microbiota composition and metabolites [102,103]. For example, a five-month nicotinamide riboside (the precursor of NAD^+^) supplement in twins discordant for body mass index could modify gut microbiota composition and improve muscle stem cell functions and mitochondrial biogenesis along with DNA methylation alterations of several genes, including metabolic genes, such as *NAPRT* and *PPARy* [104], which are also involved in redox reactions [105,106]. On the other hand, a high-fat/carbohydrate diet increased *Firmicutes (Clostridium)*, *Prevotella*, and *Methanobrevibacter* but decreased *Bacteroides*, *Bifidobacterium*, *Lactobacillus*, and *Akkermansia*, which are SCFA producers. These alterations are associated with compromised intestinal epithelial barrier integrity, increased inflammation (which induces ROS), and dyslipidemia due to a decrease in the expression of fasting-induced adipocyte factor [107]. However, a diet rich in fiber and acetate could mitigate gut dysbiosis by increasing the abundance of *Bacteroides acidifaciens* and modulating the *Firmicutes* to *Bacteroidetes* ratio in hypertensive mice [108]. De Filippis et al. also found that high adherence to the Mediterranean diet, characterized by a high intake of fruits, vegetables, legumes, nuts, and whole grains, and a moderate intake of fish and poultry, is associated with a beneficial microbiome and the interlinked metabolome profile [109]. In another study, Ghosh et al. investigated the effects of a 1-year Mediterranean diet intervention on the gut microbiome of elderly individuals across five European countries. They found that adherence to the Mediterranean diet resulted in significant positive changes in the gut microbiome, including increased microbial diversity and the enrichment of health-associated microbial taxa. The diet also led to a reduction in inflammatory markers, increased production of SCFAs, and improvements in health parameters, such as cognitive function and frailty [110]. On the other hand, ultra-processed foods and food additives can increase intestinal permeability and inflammation by altering the gut microbiome community [111]. In addition to diet, the composition of the gut microbiome (e.g., higher abundance of *Akkermansia*) affects dietary fiber metabolism, leading to the production of SCFAs and anti-ROS molecules, such as glutathione, which confer significant health benefits [112]. In contrast, reduced levels of SCFAs, like propionate, butyrate, and acetate, along with purine starvation required for glutathione synthesis have been observed in longitudinal multi-omics studies of stool samples from patients with irritable bowel syndrome [113].

Other types of dietary interventions, such as caloric restriction, could also improve energy expenditure, enrich gut microbial diversity, and alter bile acid metabolism in mice. Interestingly, microbial transfer from these mice into mice fed an obesogenic diet improves the bile acid metabolism of the recipient mice and mitigates fatty liver [114].

Probiotic and postbiotic use are other types of interventions to mitigate metabolic diseases. For instance, in high-fat diet-induced obese mice, gavage of *Bacteroides dorei* and *Bacteroides vulgatus* could attenuate weight gain by improving branched-chain amino acid catabolism in brown fat tissue, suggesting that *Bacteroides* probiotics may be helpful in the treatment of obesity [115]. In a recent meta-analysis of human studies, probiotics could also significantly reduce the blood cholesterol and lipid levels in patients with metabolic disease [116]. Another meta-analysis on the effect of *Bifidobacterium* probiotic supplementation on fasting blood glucose levels reported no effects in humans but a highly significant decrease in animal models of metabolic syndrome, type-2 diabetes, and obesity [117]. It suggests that *Bifidobacterium* alone may not affect humans’ blood glucose levels. However, another recent meta-analysis of human studies found that probiotics and synbiotics (a combination of probiotics and prebiotics) can reduce fasting blood glucose levels, body mass index, and LDL cholesterol levels in patients with metabolic syndrome [118]. A recent umbrella meta-analysis of 13 meta-analyses on patients with NAFLD also confirmed beneficial effects of probiotics, prebiotics, and synbiotics on body mass index [119]. Similarly, a meta-analysis of 97 meta-analysis studies showed beneficial effects of probiotics, prebiotics, and synbiotics on the overweight/obesity indices [120]. In type-1 diabetes, a recent meta-analysis reported that probiotic supplementation could moderately decrease fasting blood glucose levels but had no significant effect on serum HbA1c [121].

Considering postbiotics, dietary supplements containing acetate and butyrate produced by gut microbiota could improve glucose control in type 1 diabetic mice by upregulating β-cells’ functional genes and insulin production [122]. SCFA supplementation could also recapitulate the corresponding microbiota-induced chromatin modifications and gene expression alterations in germ-free mice [123]. Furthermore, in an in vitro study, vitamin B12 was shown to play a critical role in sustaining cellular DNA methylation status of genes linked to cell proliferation and intestinal barrier function, in addition to improving fatty acid and mitochondrial metabolism, while suppressing the inflammatory response of ileal epithelial cells [124].

While dietary intervention modulates gut microbiome and metabolic health in adults, maternal nutritional intervention can also help the offspring’s health status. In a study in mice, high-fat diet-induced maternal obesity increased the risk of metabolic disease in offspring. However, maternal use of phlorizin (extracted from apple tree bark or leaves) improved gut dysbiosis and increased SCFA-producing bacteria (*Akkermansia* and *Blautia*), while decreasing LPS-producing bacteria, mitigating not only maternal but also their offspring’s metabolic disease [125]. Maternal dietary genistein (an isoflavone of soybean) could also improve offspring metabolic functions, lipid panel aberrations, and glucose intolerance and decrease high-fat diet-induced body fat in offspring [126]. On the other hand, prenatal dexamethasone exposure could have harmful effects and impact gut microbiota composition (e.g., increased *Klebsiella* but decreased abundance of *Akkermansia*, *Bacteroides*, and *Parabacteroides*), as investigated in 6-month-old infants, with these changes persisting for two years in female infants. This is associated with alterations in bile acid metabolism and a higher risk of cholestatic liver injury. Mechanistic studies in rats and cell analysis revealed that this effect is due to a decrease in *Muc2* (mucin 2) gene expression mediated by GR/HDAC11 signaling activation, leading to *Cdx2* epigenetic dysregulation [127] where CDX2, a transcription factor, suppresses ROS [128]. Low-dose tetracycline treatment during the perinatal period (4 days in the embryonic period and 4 weeks postnatally) in mice also alters gut microbiota in dams, with these changes lasting into adulthood and being associated with persistent alterations in body weight and growth trajectories [129].

## 9. Dietary and Microbiome-Induced Health Benefits Mediated by Epigenetic Modifications

An earlier study in mice uncovered that gut microbiota could regulate host global histone acetylation and methylation, which appears to be diet-dependent, as, in contrast to a polysaccharide-rich diet, a Western-type diet could prevent these effects [123]. In a subsequent human study, long-term use (18 months) of a Mediterranean diet or a low-meat diet rich in polyphenols could delay epigenetic (DNA methylation) biological aging [130]. In another relatively large recent microbiome and whole-genome DNA methylome study in humans, the authors reported a correlation between the abundance of *Ruminococcus*, obesity, and altered DNA methylation of a region located between the *MACROD2* and *SEL1L2* genes, both of which are involved in cellular stress responses [131]. Furthermore, hypercholesterolemia, a component of metabolic diseases, has been linked to the upregulation of two stool small RNAs (*ID 2909606* and *ID 2149569)* correlated with the abundance of *Coprococcus eutactus* (*Lachnospiraceae* family) and *Blautia wexlerae*, respectively [132]. In another study, diet style (e.g., in vegans, vegetarians, or omnivores) not only affected gut microbiota composition but also altered the expression of 49 stool miRNAs in humans. For example, the expression of miR-636 and miR-4739, related to lipid metabolism, exhibited an inverse correlation with the duration of the non-omnivorous diet. On the other hand, 17 miRNAs were directly correlated with animal proteins and lipids. Since the abundance of *Prevotella* and *Roseburia* was higher in omnivores and the abundance of *Bacteroides* was lower vs. vegans and vegetarians, the expression pattern of miRNAs was distinctive in these three dietary groups. Additionally, the plasma and stool expression of miR-362-3p exhibited positive correlation, but let-7a-1-3p exhibited inverse correlation [133]. Table 1 shows diverse types of dietary interventions involving vitamins, nutritional compounds, and postbiotics, or the use of probiotics, prebiotics, and metabolic drugs that may modulate the gut microbiome or the epigenome of genes related to inflammation and ROS in metabolic diseases. Although some of these studies have not analyzed the influence of these interventions on both gut microbiota and the epigenome, it is expected that the research community will uncover these unknown aspects in the coming years.

## 10. Conclusions

Alterations in gut microbiota composition and, thus, function can exacerbate oxidative stress, cellular and DNA damage, inflammation, and epigenetic alterations, increasing the risk of metabolic diseases, such as insulin resistance, dyslipidemia, obesity, and hypertension. Gut dysbiosis promotes ROS production and disrupts the balance of antioxidant defense systems, initiating a vicious cycle that further promotes gut dysbiosis. Gut-derived metabolites, such as SCFAs and other microbial-derived compounds and vitamins, can modulate oxidative stress and improve metabolic function. Gut dysbiosis-induced inflammation can also disrupt gut barrier integrity and increase intestinal permeability, which allows gut-derived hazardous products to enter the liver and systemic circulation, further triggering oxidative stress, inflammation, and epigenetic alterations associated with metabolic diseases. As gut microbiota is transferred to close family members, any microbiome-related disease may resemble genetic diseases. Therefore, more scrutiny on the potential contribution of the microbiome in the pathogenesis of those familial metabolic diseases is warranted.

While gut microbiota-induced metabolic diseases could transfer to the next generation, there are specific therapeutic interventions that may mitigate metabolic diseases both in affected individuals and their offspring. In addition to current medical therapeutics, physical exercise, intermittent fasting, dietary modifications, prebiotics, probiotics, postbiotics, and fecal microbiota transplantation may alter gut microbiota composition and reduce inflammation, oxidative stress, and epigenetic alterations associated with metabolic syndrome. Future research should focus on identification of specific bacterial genera and species involved in the pathogenesis of particular metabolic diseases and on developing and optimizing therapeutic interventions to precisely target the responsible gut microbiome and its metabolic pathways. This includes identifying specific prebiotics, probiotics, and postbiotics that can effectively modulate the gut microbiota to reduce oxidative stress and inflammation. Further studies to understand the long-term effects and safety of fecal microbiota transplantation are crucial, as is exploring its potential to prevent or reverse familial metabolic diseases. Additionally, personalized approaches considering individual microbiome profiles and genetic predispositions may enhance the efficacy of these interventions. Understanding the interplay between gut microbiota, epigenetic modifications, and metabolic health will be essential in developing comprehensive strategies to mitigate the transgenerational impact of gut microbiota-induced metabolic diseases.

## Figures and Tables

**Table 1:** Various interventions that mitigate metabolic diseases by affecting gut microbiota and/or the epigenetic status.

	Intervention	Study Subjects	Gut Microbiota Alteration	Functional Changes	Mechanism	Ref.
Vitamins	Vitamin C	Humans	Increases Actinobacteria	Boosts immune functions, glucose homeostasis, and cell metabolism	DNA methylation alterations of 116 genes (13 hypo and 103 hypermethylated)	[134]
	Vitamin B12	Stem cells, in vitro	Not applicable	Boosts cell regeneration	Mediates H3K36me3 generation	[135]
	Vitamin B12 deficiency (4 weeks)	Mice with DSS (dextran sodium sulphate) challenge	No change in normal mice but altered abundance of 30 genera	Reduces DSS-induced epithelial tissue damage	Unknown	[136]
	Vitamin B12, excess amount (1000-fold)	Mice	Decreases α diversity, *Clostridia vadin* BB60 and *Lachnospiraceae* NK4A136 groups, but increases *Parasutterella*	Immune activation, production of IL-17A and the IL-12/23p40 subunit cytokines in colon	Unknown	[137]
	Folic acid and zinc	Rats (hyperuricemia, induced by a high-purine diet)	Increase the abundance of probiotic bacteria and reduce pathogenic bacteria	Improve hyperuricemia	Unknown	[138]
Postbiotics	Butyrate	Human primary liver cells	Not applicable	Increases *AhR* expression and its target genes	Unknown	[139]
	Butyrate	Humans with obesity and diet-induced obese mice	Unknown	Decreases quinolinic acid- induced BDNF suppression and improves cognition	Increases H3K18ac at *BDNF* promoter	[140]
	Acetate and butyrate	In vitro on microglia	Unknown	Reduces microglia cytokine production	Reduces HDAC activity and NF-κB nuclear translocation	[141]
	Lactate	Human cells and mouse	Macrophage exposed to lactate-producing bacteria	Enhances *Arg1* (a metabolic gene) and wound healing	Histone lactylation	[73]
	p40, a probiotic functional factor	In early life of mice	Modulate gut microbiota	Long-lasting TGFβ production by intestinal epithelial cells, expands Tregs and mitigates gut inflammation	Epigenetic increase of *TGFβ* expression by H3K4me1/3 persisting into adulthood	[142]
Prebiotics	Fermented brown vs. white rice	Patients with metabolic syndrome	Increases species belonging to the *Clostridia* class	Reduces inflammation	Increases blood SCFAs	[143]
	Inulin (a soluble fiber)	Mice and in vitro studies	Unknown	Reduces microglia TNF-α secretion	Increases gut SCFA production and its blood level	[144]
	Inulin fiber and multi-strain probiotics	High-fat/sucrose diet-induced steatohepatitis in rats.	Unknown	Improves steatosis, inflammation, liver enzymes, fibrosis, and lipid panel; decreases TGFβ1 (a fibrotic marker) and IL6	Decreases hepatic *Yap1* and miR-1205 expression, and upregulated *Lats1*, *Nf2* and lncRNA SRD5A3-AS1	[145]
	High fiber diet	Humans’ NAFLD	Potentially change gut microbiome	Reduces liver steatosis	Decreases serum SCFAs (unexpectedly)	[146]
Probiotics or fecal microbiota transplatation	Fecal microbial transplantation	Humans	Gut microbiome alterations, including *Prevotella* ASVs	Modulates plasma metabolome and the epigenome of immune cells	*Prevotella* ASVs correlated with methylation of *AFAP1* involved in mitochondrial function, and insulin sensitivity	[83]
	*Lactobacillus reuteri*	Pregnant mice	Gut microbiome alterations	Potential prevention of autism-like symptoms	Altered DNA methylation of genes linked to neuro and synaptogenesis, synaptic transmission, reelin signaling, etc. in offspring	[147]
	*Lactobacillus* suplementation	High fat diet (HFD) induced insulin-resistant rats	Altered gut microbiota composition in favor of *Lactobacillus*	Reduces hyper-glycemia, hyper-insulinemia, hyper lipidemia, and hepatic- intestinal damage	Mitigates methylation of H3K79me2 and demethylation of H3K27me3 and reduces *Foxo1* expression	[148]
Periodic fasting	5 days of periodic fasting	Humans	Increased gut microbiota diversity, *Prevotella*, *Lactobacillus*, and *Christensenella* abundance	Improves metabolism	Increases mitochondrial DNA, *SIRT1*, *SIRT3*, and miRlet7b-p expression in blood cells	[149]
Nutritional compounds	Sulforaphane	Rats	Improves gut microbial diversity and functions	Reduces uric acid level	Epigenetic modification of *Nrf2* gene	[150]
	*Saccharomyces boulardii*	DSS-induced colitis in humanized mice	Increase microbial SCFAs production	Mitigates colon damage and inflammatory responses	Modulates the cytokine profile	[151]
	Black tee	HFD feeding mice	Reverses HFD-induced gut dysbiosis	Prevents obesity	DNA methylation alterations, including imprinted genes in the spermatozoa of HFD mice	[152]
	Urolithins	HFD obese rats and mice	Modulated gut microbiota and in mice increase population of *Akkermansia* spp.	Decreases body weight, inflammation, ROS, insulin resistance and restores serum lipid profile	Unknown	[153,154]
	Policaptil Gel Retard	HFD feeding mice	Increases gut *Bacteroidetes* and decreases *Firmicutes*	Decreases food intake and body weight, improves metabolic state	Modulates expression of metabolic genes and rescues *Igfbp2* expression	[155]
Prescribed drugs	Metformin, oral	Mice	Increases SCFA- producing microbes like *Lachnospiraceae, Alistipes*, and *Ruminococcaceae*	Decreases colon adenocarcinoma proliferation	Increases circulating propionate and butyrate	[156]
	Metformin	ob/ob mice (genetically modified obese mice)	Reduces *Bifidobacterium* and increases *Akkermanisia muciniphlia* proportion	Increases tauroursodeoxycholic acid, which reduces ROS and intestinal inflammation	Tauroursodeoxycholic acid blocks KEAP1 binding to Nrf2, leading to Nrf2 nuclear translocation, initiating antioxidant gene expression	[157]
